# Seasonal infestation patterns of ticks on Hokkaido sika deer (*Cervus nippon yesoensis*)

**DOI:** 10.1017/S0031182024001227

**Published:** 2024-10

**Authors:** Kotaro Shimizu, Michito Shimozuru, Masami Yamanaka, Genta Ito, Ryo Nakao, Toshio Tsubota

**Affiliations:** 1 Laboratory of Wildlife Biology and Medicine, Faculty of Veterinary Medicine, Hokkaido University, Sapporo, Japan; 2 Shiretoko Nature Foundation, Shari-gun, Japan; 3 Laboratory of Parasitology, Faculty of Veterinary Medicine, Hokkaido University, Sapporo, Japan

**Keywords:** attachment site preferences, co-feeding transmission, co-occurrence, *Haemaphysalis* spp, Hokkaido sika deer, interspecies interactions, intraspecies interactions, *Ixodes* spp, tick, tick–host interactions

## Abstract

Ticks prefer specific feeding sites on a host that are influenced by host–tick and tick–tick interactions. This study focused on the spatiotemporal distribution of ticks in Hokkaido sika deer, an important tick host in Hokkaido, Japan. Tick sampling was performed on the sika deer in the Shiretoko National Park between June and October 2022. Ticks were collected from 9 different body parts of the deer to compare their attachment site preferences. Interspecific and intraspecific relationships among ticks were examined using co-occurrence analysis. The collected ticks were nymphal and adult stages of 4 species: *Ixodes ovatus*, *Ixodes persulcatus*, *Haemaphysalis japonica* and *Haemaphysalis megaspinosa*. Seasonal variations in tick burden were observed, with *I. persulcatus* and *I. ovatus* peaking in June and declining towards October; *H. japonica* showing low numbers in July and August and increasing from September; and *H. megaspinosa* appearing from September onwards with little variation. Attachment site preferences varied among species, with a significant preference for the pinna in *I. ovatus* and *I. persulcatus*. *Haemaphysalis japonica* was mainly found on the body and legs between June and August, and shifted to the pinna from September. *Haemaphysalis megaspinosa* showed a general preference for areas other than the legs. Co-occurrence analysis revealed positive, negative and random co-occurrence patterns among the tick species. Ticks of the same genus and species exhibited positive co-occurrence patterns; *I. ovatus* showed negative co-occurrence patterns with *Haemaphysalis* spp. This study revealed the unique attachment site preferences and distinct seasonal distributions of tick species in the Hokkaido sika deer.

## Graphical abstract

**Figure:**
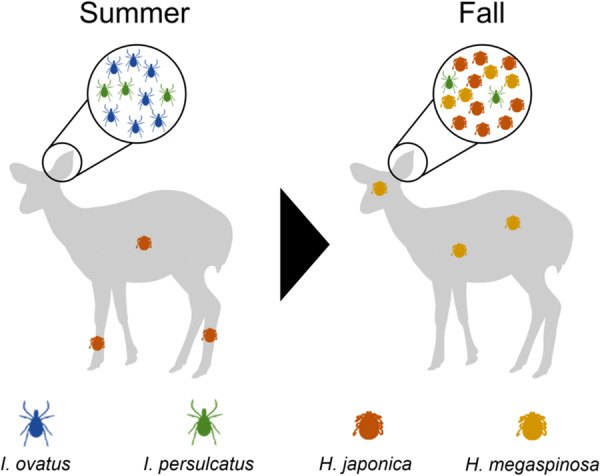

## Introduction

Most ectoparasites, including ticks, do not infest their hosts randomly but feed preferentially on specific body parts (Andrews *et al*., 1981; Chilton *et al*., 1992; Kiffner *et al*., 2011; Baer-Lehman *et al*., 2012; Mysterud *et al*., 2014; Lydecker *et al*., 2019; Tiffin *et al*., 2021). The distribution patterns depend on the characteristics of both the host and the tick (Kar *et al*., 2013). For example, it has been reported that many tick species prefer sites where they can effectively feed, such as the hairless parts of rodents (Pilosof *et al*., 2012). Feeding efficiency is influenced by the anatomical characteristics of the host and the morphology of the ectoparasite, that is, the compatibility of the feeding apparatus of the parasite and the feeding site on the host. Ectoparasites with short proboscises select body parts with relatively thin epidermis and capillaries close to the body surface, whereas ectoparasites with relatively long proboscises select body parts with a thicker epidermis and deeper capillaries (Pilosof *et al*., 2012). It is also important for ticks that the animal's mouth or claws are out of reach to prevent their removal during feeding. Host defence efforts and efficiency vary by body site (Reiczigel and Rózsa, 1998), and it has been reported that many ticks that feed on birds prefer their heads as feeding sites because they are difficult to be removed by grooming by the host (Fracasso *et al*., 2019).

Another important factor affecting the spatiotemporal distribution of ticks is interspecific interactions. Such interspecific interactions include cases where 1 parasitic species promotes or inhibits infestation by another species (Fenton *et al*., 2010; Hellard *et al*., 2015). For example, some hard ticks (Acari: Ixodidae) that feed on the eastern rock sengis (*Elephantulus myurus*) may facilitate the invasion of other tick species onto the same host (no records of attachment sites) (Lutermann *et al*., 2015). In contrast, *Ixodes scapularis* and *Dermacentor albipictus* have been reported to compete for preferred attachment sites on white-tailed deer (*Odocoileus virginianus*) (Fellin and Schulte-Hostedde, 2022). In addition to interspecific relationships, facilitative interactions within the same tick species have been reported, possibly due to the decreased immune response of the host as the tick infestation burden increases (Lutermann *et al*., 2015).

Understanding tick infestation patterns in host animals is important for efficient tick sampling, effective treatment of animals with repellents and acaricides, and, consequently, control of tick-borne diseases (TBDs) (Schmidtmann *et al*., 1998). Hokkaido sika deer (*Cervus nippon yesoensis*), a subspecies of the sika deer (*Cervus nippon*), is one of the most important hosts for several tick species of the genera *Ixodes* and *Haemaphysalis* (Tsukada *et al*., 2014; Elbaz *et al*., 2020; Iijima *et al*., 2022). The population of sika deer in Hokkaido has increased over the last 20–30 years (approximately 670 000 between 2014 and 2020) (Ikeda and Koizumi, 2024). Because deer are known carriers of several tick-borne pathogens such as Piroplasmida and *Anaplasma* (Lee *et al*., 2014; Elbaz *et al*., 2017), it is of critical importance to understand the spatiotemporal distribution of ticks on Hokkaido sika deer.

The aim of this study was to understand tick infestation patterns in Hokkaido sika deer. More specifically, the research focused on seasonal differences in infestation patterns, preferred attachment sites for each tick species and interspecific interactions between different tick species.

## Materials and methods

### Study area

This study was conducted in the Rusha area of the Shiretoko Peninsula, eastern Hokkaido, Japan (44°11′N, 145°11′E). The area from the centre of the peninsula to Shiretoko Cape, including the research site, is designated Shiretoko National Park and is a UNESCO World Heritage Site where hunting is prohibited. This area is a narrow estuary coast that stretches from south to north for approximately 3 km, and its climate is classified as Subarctic. Climate information for the sampling period in Utoro (44°03′N, 144°59′E), the closest climate observation point to the study area, is shown in Table 1. Based on the definitions provided by the Japan Meteorological Agency, March–May is defined as spring, June–August as summer, September–November as autumn and December–February as winter. Six native mammals, namely the Hokkaido sika deer, Hokkaido brown bear (*Ursus arctos yesoensis*), red squirrel (*Sciurus vulgaris*), red fox (*Vulpes vulpes*), raccoon dog (*Nyctereutes procyonoides*) and sable (*Martes zibellina*), have been identified in the northwestern part of the national park, including the study area (Kawamura *et al*., 2022).

**Table 1.** Climate information for the sampling period in Utoro, Japan, the closest climate observation point to the study area

	Precipitation (mm)		Temperature (°C)		
Month	Total	Highest day	Daily average	Daily high	Daily low
June	127.5	29.5	12.6	17.0	9.0
July	80.0	28.5	19.0	24.1	14.8
August	157.5	48.5	19.6	24.5	15.2
September	107.5	48.5	16.9	22.6	11.8
October	130.5	38.0	10.7	15.3	6.5

The data are provided by the Japan Meteorological Agency, Japan.

### Sampling

**Figure 1.**
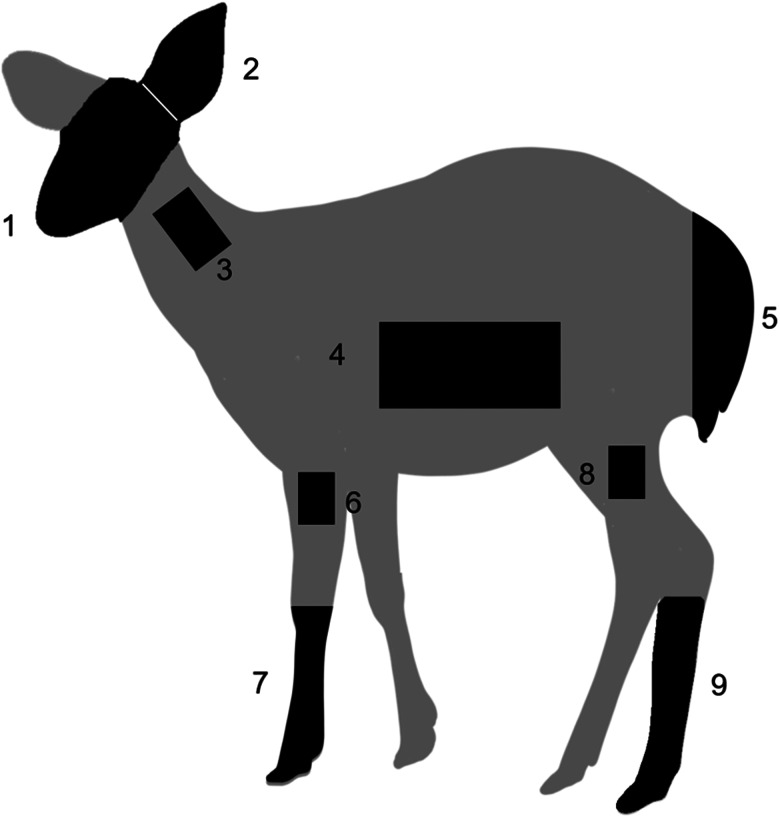
Diagram of the examined sites on the body of the sika deer: (1) head, (2) pinna, body (3: neck 50 × 70 mm^2^, 4: body side 100 × 150 mm^2^ and 5: anus and tail) and legs (6: elbow 50 × 70 mm^2^, 7: forelimb, 8: knee 50 × 70 mm^2^ and 9: hind limb); each part was examined only on the left side.

Deer were captured 48 times for tick collection between June and October 2022, totalling 37 deer, 9 of which were captured more than once. Deer capture and sampling were conducted for 3–4 days each month during the study period. The deer were anaesthetized with 0.1 mg kg^−1^ medetomidine (Dorbene Vet, Kyoritsu Seiyaku, Co., Tokyo, Japan) using a carbon dioxide-powered gun (Dan-Inject ApS, Borkop, Denmark) for intramuscular injection and awakened with 0.1 mg kg^−1^ atipamezole (Atipame, Kyoritsu Seiyaku, Co., Tokyo, Japan) injected intramuscularly after all procedures were completed. The design of the capture process was based on previous studies (Onuma *et al*., 2005). Deer age was estimated based on tooth eruption and replacement (Ohtaishi, 1977). The sample population consisted of the following age and sex structure: two 1-year-old females, one 2-year-old male, eight 2-year-old females and 26 females aged ⩾3 years. All captured deer were tagged with earmarks for identification, and individual deer were not captured more than twice in the same month to ensure that recapture did not affect sampling. Ticks were collected from the head, pinna, body (neck: 50 × 70 mm^2^, body side: 100 × 150 mm^2^ and anus tail) and legs (elbow: 50 × 70 mm^2^, forelimb, knee: 50 × 70 mm^2^ and hindlimb) of the sika deer; only the left half of the body was examined (Fig. 1). Ticks found on the host were recorded as either feeding or questing. Owing to the extremely low number of questing ticks (only a few *Ixodes* spp. males), no distinction between the feeding and questing states was made in subsequent data analyses. The sampling sites were chosen based on previous studies (Matthee *et al*., 1997; Kiffner *et al*., 2011; Mysterud *et al*., 2014). The collected ticks were identified by species, sex and life stage based on their morphological characteristics (Yamaguti *et al*., 1971) using a stereomicroscope. Due to the difficulty in accurately identifying larvae morphologically, they were not included in the analysis.

### Statistical analysis

The abundance and prevalence of each tick species attached to each deer per month were determined. Tick abundance was defined as the mean number of each tick species attached per deer per month, whereas tick prevalence was defined as the proportion of infested individuals among the captured deer. The monthly mean number of ticks per body part was calculated to determine the body parts of the deer that were preferred for tick attachment. The number of ticks on different body parts was compared using the Kruskal–Wallis test (Vargha and Delaney, 1998), with 3 degrees of freedom. Subsequent post-hoc analysis was conducted using the Steel–Dwass method for multiple comparison tests (Neuha and Bretz, 2001). The Kruskal–Wallis test and Steel–Dwass post-hoc analysis were performed separately for each tick life stage (males, females and nymphs) as well as for the total number of ticks across all stages, to compare tick distributions among different body parts. Because most of the captured deer were females aged ⩾3 years, comparisons of tick infestation patterns by deer sex or age were not made. All statistical analyses were performed using R version 4.1.0 (R Core Team, 2021), and differences were considered statistically significant at *P* < 0.05. The co-occurrence patterns of different tick species and stages across attachment sites (each body part of the deer) were investigated to evaluate the inter- and intraspecific relationships between ticks. The ‘co-occurrence’ package in R (Griffith *et al*., 2016) was used to conduct a pairwise co-occurrence analysis of the species for this analysis, determining whether the co-occurrences were positive, negative or random. This package uses an algorithm that calculates the observed and expected frequencies of co-occurrence between each pair of species and stages based on the presence/absence data across a collection on each body part. These associations were interpreted as negative or positive co-occurrences when the co-occurrence frequencies were much lower or higher than expected, respectively. In this study, 4 body parts (head, pinna, body and legs) were examined for each of the 48 host individuals, resulting in a total of 192 observations (48 individuals × 4 body parts).

Pairs with expected co-occurrence numbers of <1 were excluded from the analysis to minimize the likelihood of spurious results.

## Results

All captured deer were infested with at least 1 tick, and 3026 ticks were collected during the study period. The overall composition of the tick population from 48 samplings of sika deer was 21.9% female (663/3026), 52.0% male (1574/3026) and 26.1% nymphs (789/3026). The tick species identified were *Ixodes ovatus* (*n* = 441), *Ixodes persulcatus* (*n* = 538), *Haemaphysalis japonica* (*n* = 1793) and *Haemaphysalis megaspinosa* (*n* = 254) (Table 2). The stage composition of attached ticks varied by species, with *I. ovatus* (males: 51, females: 379 and nymphs: 11), *H. japonica* (males: 1365, females: 135 and nymphs: 293) and *H. megaspinosa* (males: 134, females: 113 and nymphs: 7) being the predominant adults, whereas *I. persulcatus* (males: 24, females: 36 and nymphs: 478) were predominant at the nymphal stage (Table 2).

**Table 2.** Number of ticks collected from Hokkaido sika deer from June to October 2022

Sampling month		Jun.	Jul.	Aug.	Sep.	Oct.	Total
No. of deer sampled		11	10	10	10	7	48
Species	Stage						
*Ixodes ovatus*	Male	28	13	8	2	0	51
	Female	227	120	23	9	0	379
	Nymph	1	0	8	1	1	11
	Subtotal	256	133	39	12	1	441
*Ixodes persulcatus*	Male	12	5	2	2	3	24
	Female	23	4	2	3	4	36
	Nymph	121	122	147	63	25	478
	Subtotal	156	131	151	68	32	538
*Haemaphysalis japonica*	Male	76	24	0	344	921	1365
	Female	53	30	8	34	10	135
	Nymph	72	39	57	63	62	293
	Subtotal	201	93	65	441	993	1793
*Haemaphysalis megaspinosa*	Male	0	0	0	49	85	134
	Female	0	0	0	76	37	113
	Nymph	0	0	0	1	6	7
	Subtotal	0	0	0	126	128	254
Total		613	357	255	647	1154	3026

**Figure 2.**
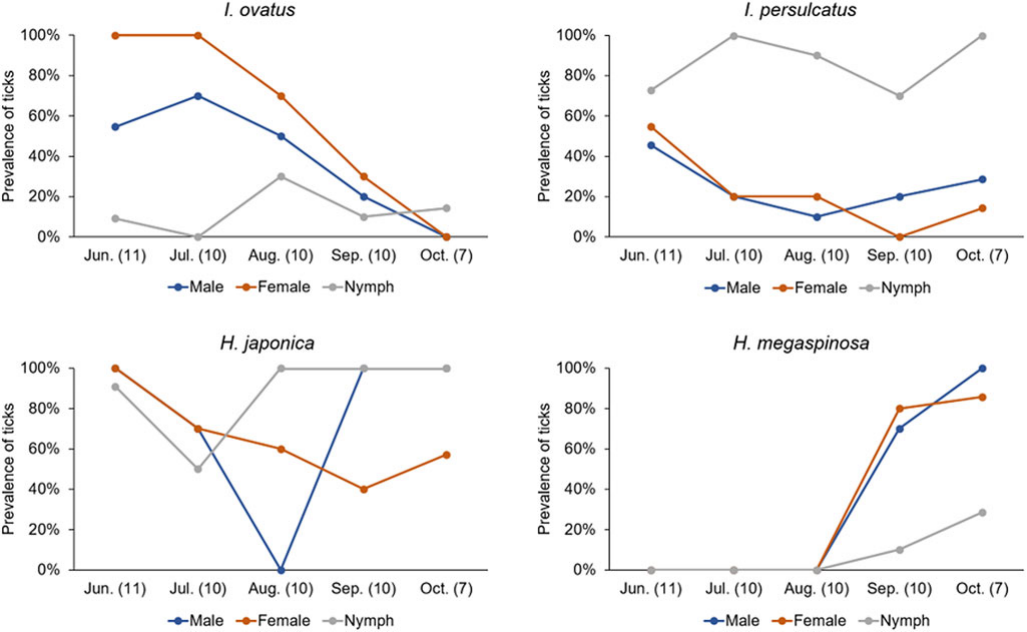
Seasonality of the prevalence of each tick species and their developmental stages parasitizing sika deer from June 2022 to October 2022. The horizontal axis represents the sampling month (number of deer).

Monthly variation in tick infestation showed different patterns for each species (Table 2 and Fig. 2). The highest infestation by *I. ovatus* was observed in June (*n* = 256), followed by that in July (*n* = 133), August (*n* = 39), September (*n* = 12) and October (*n* = 1). Infestations of *I. persulcatus* nymph were relatively consistent throughout the sampling period, with a gradual decrease from June to October (June: *n* = 156; July: *n* = 131; August: *n* = 151; September: *n* = 68; October: *n* = 32). In contrast, the number of *H. japonica* decreased from June to August (June: *n* = 201; July: *n* = 93; August: *n* = 65) but increased rapidly from September (*n* = 441) to October (*n* = 993). *Haemaphysalis megaspinosa* was not observed in June, July or August but appeared in September (*n* = 126) and October (*n* = 126).

The peak prevalence of *I. ovatus* and *H. megaspinosa* was observed in the same month as their peak abundances (Table 2 and Fig. 2). The prevalence of *I. persulcatus*, similar to its abundance, showed little monthly variation and was always higher than 70%. The trend in the prevalence of *H. japonica* was different from that of the other 3 species, with a high prevalence from June to August, even when the abundance was relatively low compared to September to October (June: 90%, July: 80%, August: 100%).

**Figure 3.**
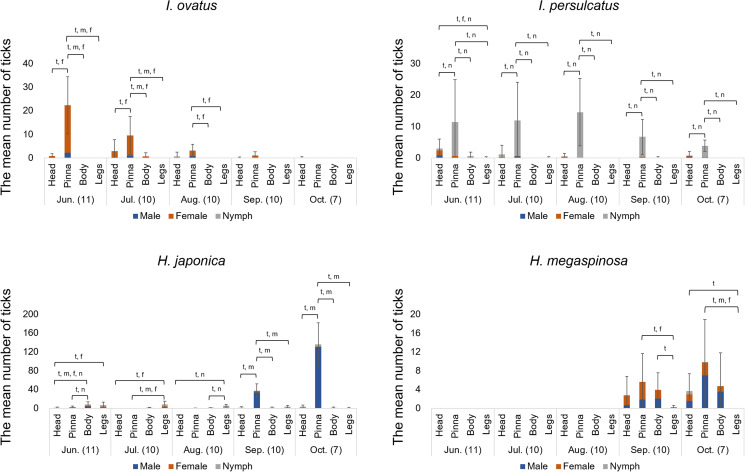
Seasonal occurrence of ticks on the examined attachment sites of infested sika deer from June 2022 to October 2022. Error bars represent standard errors of the total number of ticks attached to each site, and the horizontal axis represents the sampling month (number of deer). (t, m, f, n: *P* < 0.05, based on the Steel–Dwass method for total, male, female and nymph ticks, respectively.).

This study revealed that the preference for the body parts to which ticks attach varies significantly depending on the tick species (Fig. 3). In *I. ovatus* adults, significantly higher numbers were observed on the pinna than on other body parts from June to August (June: *H*-statistic = 36.0; July: *H*-statistic = 20.8 and August: *H*-statistic = 18.4). Similarly, *I. persulcatus* nymphs were significantly more abundant in the pinna, and this trend was observed throughout the sampling period, whereas *I. persulcatus* adults showed a slight preference for the head (June: *H*-statistic = 17.5; July: *H*-statistic = 29.6; August: *H*-statistic = 27.9; September: *H*-statistic = 25.9; October: *H*-statistic = 20.8). Conversely, *H. japonica* exhibited notable monthly variations in attachment sites. It was primarily found on the body and legs in June, July and August (June: *H*-statistic = 19.3; July: *H*-statistic = 18.0; August: *H*-statistic = 28.4); however, a significant shift was observed in September and October, with a marked aggregation on pinnae across all stages (September: *H*-statistic = 25.5; October: *H*-statistic = 17.1). This trend was observed for both nymphs and adults, but only the total number of all stages and the number of males showed statistically significant differences between September and October. In contrast to the other 3 species, which were highly aggregated on specific body parts, *H. megaspinosa* was widely distributed on the head and body, with preference for the pinna (September: *H*-statistic = 9.2; October: *H*-statistic = 12.8).

**Figure 4.**
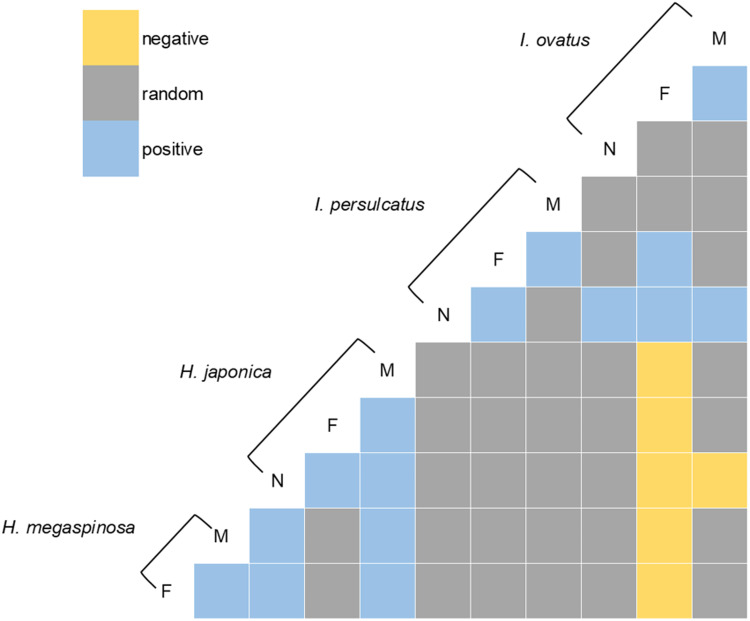
Heat map representing positive, negative and random associations of tick species/stages found in each body part of individual sika deer, as determined by the stochastic co-occurrence model in the ‘co-occurrence’ package in R. Species names and stages are arranged to show columns and rows representing pairwise relationships with other species/stages (M; male, F; female, N; nymph).

In the analysis of co-occurrence patterns among ticks, 66 pairs of combinations derived from different tick species and stages were considered. Consequently, 13 pairs were removed from the analysis because their expected co-occurrence was <1, leaving 53 pairs for further analysis. Positive (28%, 15/53), negative (11%, 6/53) and random co-occurrence patterns (60%, 32/53) were observed (Fig. 4). Ticks of the same species and genus tended to exhibit positive co-occurrence patterns. In particular, positive co-occurrence patterns between females and males of the same species were observed in all species and between females and nymphs in *I. persulcatus* and *H. japonica*. Conversely, females of *I. ovatus* showed negative co-occurrence patterns with all stages of *H. japonica* and both males and females of *H. megaspinosa*.

## Discussion

This is the first detailed study of tick infestation patterns in the Hokkaido sika deer. Seasonal activity, attachment site preferences and co-occurrence patterns of tick species were investigated. In the current study, 4 tick species, *I. ovatus*, *I. persulcatus*, *H. japonica* and *H. megaspinosa*, were collected from Hokkaido sika deer, and each species showed unique seasonal fluctuations. Specific attachment site preferences were observed for each tick species, with *H. japonica* showing seasonal variation in attachment site preferences. Ticks showed various co-occurrence patterns on the body surfaces of deer, suggesting the presence of inter- and intraspecific interactions between ticks and their attachment sites. These results provided robust evidence that complex host–tick and tick–tick interactions exist during the selection of tick attachment sites, as previously suggested (Andrews *et al*., 1981; Chilton *et al*., 1992; Kiffner *et al*., 2011; Baer-Lehman *et al*., 2012; Mysterud *et al*., 2014; Lydecker *et al*., 2019; Tiffin *et al*., 2021).

Adults of *I. ovatus* were collected in the highest numbers in June and then declined towards October. Previous studies on the seasonal activity of *I. ovatus* in the natural environment of Honshu, Japan reported that *I. ovatus* was highly active from April to mid-July but declined markedly in late-August (Fujimoto, 1996, 1997). Although sampling prior to June was not possible in this study, the activity pattern of *I. ovatus*, with a gradual decline beginning in June, was consistent with the activity pattern of this species reported in previous studies. In contrast, nymphs peaked in abundance and prevalence in August, but the number collected was very small throughout the study period. In general, larvae and nymphs of 2- and 3-host ticks prefer smaller animals, whereas adults prefer larger animals (McCoy *et al*., 2013). Given that several small- and medium-sized animal species are present in the study area (Kawamura *et al*., 2022), it is likely that other animal species serve as primary hosts for *I. ovatus* nymphs. This preference for smaller hosts likely contributed to the low number of nymphs collected from the deer in this study.

In contrast to the other 3 species that displayed clear seasonal variation, *I. persulcatus* abundance remained stable throughout the study period. A study in Finland reported that *I. persulcatus* nymphs survived for long periods without exhibiting clear seasonal fluctuations (Pakanen *et al*., 2020), which is consistent with the results of the current study. Notably, nymphs were predominant throughout the sampling period, and adult ticks were present in very small numbers. As mentioned above, it is well known that nymphal ticks prefer small- to medium-sized mammals as hosts, while adult ticks tend to parasitize larger mammalian species (McCoy *et al*., 2013). However, the scarcity of *I. persulcatus* adults in this study suggested that there are other suitable hosts for these adults in Hokkaido. The most likely host of *I. persulcatus* adults is the Hokkaido brown bear, another large mammal present in Hokkaido. A previous study examining ticks infesting Hokkaido brown bears reported that most *I. persulcatus* infesting bears were adult ticks (Ozawa and Kadosaki, 1996). However, it should be noted that the previous and current studies have been conducted in different areas of Hokkaido (Ozawa and Kadosaki, 1996). Further investigations involving multiple wild animals at the same location are necessary to gain a better understanding of the host preferences of *I. persulcatus*.

The total number of *H. japonica* was low in summer but increased significantly in autumn. Furthermore, the increase in number from September onwards was primarily due to the presence of males, whereas the numbers of females and nymphs did not show such significant fluctuations. A similar seasonal variation was observed for *H. megaspinosa*, which was collected only from September onwards, with males predominating in October. A previous study on Hokkaido brown bears reported that the abundance of *H. japonica* and *H. megaspinosa* increased from November, which is somewhat different from the results of the current study (Ozawa and Kadosaki, 1996). Although geographic and temporal gaps between this and previous studies should be noted, these differences in results suggested that ticks establish different relationships with different animal species. The male-biased attachment in *Haemaphysalis* spp. observed in the current study has also been previously reported and may be related to reproductive strategies (Ozawa and Kadosaki, 1996; Tsunoda, 2012). Metastriate ticks, including *Haemaphysalis* spp., secrete pheromones that control aggregation, mating and ejaculation after attachment to the host, and they mate only on the body surface of the host, whereas *Ixodes* spp. frequently mate before attachment to the host (Kiszewski *et al*., 2001). Therefore, it is advantageous for males of *Haemaphysalis* spp. to attach to their hosts earlier than females, wait for mates and remain on the host longer after mating (Tsunoda, 2012). Another possibility is that unmated males attempt to overwinter on the host (Ozawa and Kadosaki, 1996; Doi *et al*., 2018); however, research on the overwintering strategies of ticks is limited (Kim *et al*., 2020), and no conclusive statements can be made.

Co-occurrence analysis revealed positive co-occurrence patterns between male and female ticks of the same species, and these patterns were observed in all tick species. These results suggested that the reproductive strategy of ticks promotes male–female aggregation, as previously reported (Sonenshine, 1985, 2004). Additionally, positive co-occurrence patterns between females and nymphs were observed for *I. persulcatus* and *H. japonica*. Differences in the attachment sites at different life stages have been reported in several species (Tiffin *et al*., 2021). Indeed, *I. persulcatus* nymphs were clustered on the pinna, whereas adults were attached to both the pinna and head, with a slightly higher frequency of attachment to the head. However, these two stages shared the same attachment sites on the head and pinna, possibly resulting in a positive co-occurrence pattern. These results are interesting when considering disease dynamics in nature *via* co-feeding transmission. Co-feeding transmission occurs when pathogens are transmitted between infected and naïve vectors that feed in close temporal and spatial proximity to hosts that have not yet developed systemic infections (Belli *et al*., 2017). Co-feeding transmission has been confirmed in several animals and tick species for some pathogens, such as tick-borne encephalitis virus (Labuda *et al*., 1993, 1996) and *Borrelia burgdorferi* (Gern and Rais, 1996; Ogden *et al*., 1997; Gern *et al*., 1998; Randolph *et al*., 2003). Both pathogens are vectored by *I. persulcatus* in Hokkaido (Hayasaka *et al*., 1999; Muto *et al*., 2015; Okado *et al*., 2021). The results of this study suggested that similarities in attachment site preference between ticks at different stages could potentially facilitate co-feeding transmission. Hence, this study has important implications for understanding the dynamics of TBDs in wildlife and highlights the need for further research to understand how ticks of different species and stages interact with each other. The results of the co-occurrence analysis of different species showed negative co-occurrence patterns between *I. ovatus* and *Haemaphysalis* spp. Three hypotheses may explain the positive and negative co-occurrence patterns of ticks among different species.

The first hypothesis was that similarities and differences in phenology between the same or different genera and species affect co-occurrence patterns. Indeed, previous studies have reported phenological differences between *Ixodes* spp. and *Haemaphysalis* spp. and similarities within the same genus (Ozawa and Kadosaki, 1996; Kanamori *et al*., 2009). In the current study, *I. ovatus* and *H. megaspinosa* peaked in abundance and infestation rates in summer and autumn, respectively. A previous study also reported clear phenological differences between the two species (Ozawa and Kadosaki, 1996). Based on these results, phenological differences may be the most plausible explanation for the negative co-occurrence patterns observed between the two species.

The second hypothesis was that morphological similarities within the same taxon promote positive co-occurrence among tick species of the same genus. A previous study on similar attachment site preferences within the same subgroups of ectoparasites supports this hypothesis (Pilosof *et al*., 2012). The most prominent morphological difference between *Ixodes* spp. and *Haemaphysalis* spp. is the length of their mouthparts, which consists of a pair of chelicerae and a single hypostome. *Ixodes* spp. have longer and sharper mouthparts than *Haemaphysalis* spp. and secrete fewer cementing substances to hold the body in place during engorgement (Balashov, 1972). Such morphological differences could influence the availability of host blood vessels and resistance to grooming (Pilosof *et al*., 2012) and may explain the similarities in attachment site preferences among the same genus groups. The fact that *I. ovatus* adults and *I. persulcatus* nymphs were primarily attached to the pinna during the study period suggests a high degree of compatibility with the pinna. The pinna has been reported to be the preferred feeding site for certain ticks that feed on ungulates because it is difficult for the host to groom this area with its mouth and legs (Matthee *et al*., 1997; Handeland *et al*., 2013; Mysterud *et al*., 2014). In contrast, the distribution of *H. megaspinosa* was wider than that of the other tick species found in the current study and was distributed on the head, pinna and body. Similar attachment patterns have been observed in other tick species (Sundstrom *et al*., 2021; Tiffin *et al*., 2021). This suggested that some species may have adapted to a wide range of host body parts. However, the negative co-occurrence patterns of *I. ovatus* and *H. japonica* could not be explained by phenological and morphological differences alone. This is because, although both species had a high prevalence in June and July, the attachment sites were separate, and *H. japonica* showed dynamics such that it moved to the pinna from September onwards when *I. ovatus* was no longer present. This suggested that other factors play a role in the co-occurrence patterns.

The third hypothesis was that negative co-occurrence patterns were formed to avoid interference due to similarities in semiochemical communication, which may explain the patterns of *I. ovatus* and *H. japonica*. Semiochemical-mediated changes in tick behaviour and intra- and interspecific communication are well known (Sonenshine, 2004), and in some cases, some species use the same chemicals. For example, it has been reported that *Ixodes ricinus* and *I. scapularis* exhibit similar arrest behaviours by guanine and xanthine (Sonenshine, 2004). In metastriate ticks, differences in the concentrations of 2,6-dichlorophenol induce different reactions depending on the species (Sonenshine, 1985). Furthermore, when reptile ticks, such as *Bothriocroton hydrosauri*, *Amblyomma albolimbatum* and *Amblyomma limbatum* infest the same host, the movement of males searching for sexually receptive females is constrained, and males display courtship behaviour towards females of different species (Andrews *et al*., 1982*a*). It has been suggested that such signal interference may promote spatial separation among tick species (Andrews *et al*., 1982*b*; Chilton *et al*., 1992). Although there have been no previous reports on the interference of semiochemical communication between *I. ovatus* and *H. japonica*, this is the first study to suggest an interspecific interaction between these two species at the field level. Laboratory-based behavioural studies are necessary to investigate the semiochemical-mediated relationship between these two species.

Random co-occurrence was the most frequently observed among all the co-occurrence patterns. It is well known that ticks of different species feed on the same host (Baer-Lehman *et al*., 2012; Gómez-Rodríguez *et al*., 2015; Cayol *et al*., 2018). Therefore, it is not surprising that the neutral interspecific relationships suggested by the random co-occurrence patterns were most frequently observed. Neutral interspecific relationships are beneficial when sufficient resources (hosts) are available for tick survival (Canard *et al*., 2012; Butler *et al*., 2021). The tick species collected in this study are generalists that feed on various hosts (Ozawa and Kadosaki, 1996; Sashika *et al*., 2011; Tsunoda, 2012; Elbaz *et al*., 2020). Furthermore, sika deer are bigger than other land mammals (the average was 76.2 kg in this study), providing ticks with a large feeding area and abundant blood meals (Iijima *et al*., 2022), and deer density in the study area has been reported to be very high (Uno *et al*., 2022). Therefore, it is likely that ticks have sufficient feeding resources in the study area and that a neutral relationship prevailed.

This study suggested that each tick species has unique attachment site preferences on the Hokkaido sika deer body surface and positive, negative or random co-occurrence patterns with ticks of both conspecific and heterospecific groups. However, it is important to note that the observed interactions between tick species might be the result of various confounding factors. The risk of inferring species interactions from observational data has been previously discussed. Confounding effects may occur at the host individual (host age and sex), population (host sex ratio, age structure and density), parasite (presence of risk factors shared by two or more parasites) or landscape level (geological and climatic factors) (Hellard *et al*., 2015). In particular, it was not possible to examine how deer host characteristics, such as sex and age affect tick infestation. Nonetheless, this is the first study to suggest the possibility of tick species interactions on wild sika deer, highlighting the need for further experimental studies to elucidate the underlying mechanisms.

## Data Availability

The data that support the findings of this study are available from the corresponding author upon reasonable request.
